# Effects of the Replacement of Co with Ni on the Microstructure, Mechanical Properties, and Age Hardening of AlCo_1−x_CrFeNi_1+x_ High-Entropy Alloys

**DOI:** 10.3390/ma14102665

**Published:** 2021-05-19

**Authors:** Che-Fu Lee, Tao-Tsung Shun

**Affiliations:** Department of Materials Science and Engineering, Feng Chia University, Taichung 407, Taiwan; p0100064@o365.fcu.edu.tw

**Keywords:** high-entropy alloy, microstructure, mechanical properties, age hardening

## Abstract

In this study, effects of the replacement of Co with Ni on the microstructure, mechanical properties, and age hardening of high-entropy alloys of AlCo_1−x_CrFeNi_1+x_ (x = molar ratio; x = 0, 0.5, 1, denoted as X_0_, X_0.5_, and X_1_, respectively) were investigated. These three alloys exhibited a dendritic structure comprising an ordered BCC matrix, a BCC phase, and an FCC or an ordered FCC phase. From X_0_ to X_1_ alloys, the yield stress and compressive stress decreased from 1202 and 1790 MPa to 693 and 1537 MPa, respectively. However, fracture strain increased from 0.15 to 0.42. Peak age hardening at 600 °C for the X_0_ alloy was due to the precipitation of the (Cr,Fe)-rich σ phase. Peak age hardening for the X_0.5_ and X_1_ alloys was observed at 500 °C because of the precipitation of the σ phase and BCC phase, respectively.

## 1. Introduction

The concept of “high-entropy alloys (HEA)”, which was originally proposed Yeh et al. [1], provides an unresearched domain for the development of alloys. HEAs comprise 5 to 13 principal metallic elements, with the concentrations of each element being greater than 5 at.%, and ΔS_conf_ ≥ 1.5R [2]. From the very beginning, an AlCoCrCuFeNi system has been systematically investigated; this serves as a fundamental for future advances [3,4,5,6,7,8,9,10,11,12]. Due to the severe segregation of the Cu-rich FCC phase at the interdendrite, corrosion resistance decreases; as a result, the development of the AlCoCrFeNi system by the removal of Cu has been investigated [5,13]. The as-cast AlCoCrFeNi alloy exhibited a microstructure with a spinodal decomposition structure comprising an ordered BCC (B2) phase and a wall-shaped BCC (A2) phase [13]. Munitz et al. [14] revealed microstructure variations of the AlCoCrFeNi alloy after heat treatment. The as-cast alloy exhibited a dendritic morphology with a spinodal decomposition (B2 + A2) structure in the dendrite and interdendrite. The as-cast structure with the dendritic morphology did not change with heat treatment. However, different phase transformations occurred after heat treatment. Heat treatment between 650 and 975 °C led to the transformation of the BCC phase to the σ phase, resulting in a further increase in hardness. After heat treatment from 850 to 1100 °C, the FCC phase was observed. Heat treatment at 1100 °C led to the transformation of the σ phase to the BCC phase and softening of the alloy. Heat treatment at 1200 °C led to the dissolution of the FCC phase, thereby enabling the re-entry of the alloy into the miscibility gap and decomposition to a B2 matrix, with the precipitation of BCC in the dendrite core and interdendritic regions. Moreover, because of the excellent compressive properties of the AlCoCrFeNi alloy, including a yield stress of 1251 MPa, a compressive stress of 2004 MPa, and a fracture strain of 32.7%, it demonstrates immense potential for development into structural materials [15]. In addition, the multi-phase refining of the suction-cast AlCoCrFeNi alloy was proposed as a promising structural material with considerable ductility without the considerable expense of strength by feasible thermomechanical processing [16]. The wear rate of the AlCoCrFeNi alloy is low under high load because of the high hardness of the B2 matrix, indicative of good wear resistance for this alloy and the possibility of developing a wear-resistant material [17]. Lu et al. [18] reported excellent oxidation resistance for the AlCoCrFeNi powder subjected to heat treatment at 900–1100 °C, which is attributed to the formation of an exclusive Al_2_O_3_ scale and the inward oxygen diffusion mechanism; these results suggest that this alloy exhibits high-temperature resistance to oxidation.

Co is well known to be the most expensive element in the AlCoCrFeNi alloy, with the cost of Ni being one-third that of Co. Recently [19], with the decrease in the Co content of the AlCoCrFeNi alloy, the molar ratio of Co has decreased because of the improved compressive stress. However, the fracture strain is sacrificed because of the increase in the volume fraction of the BCC phase; this indicates that the reduction in cost only by decreasing the Co content causes alloy embrittlement. A previous study [20] has reported that Ni and Co are stronger FCC stabilizers in HEAs, with an FCC equivalent of Ni_FCC_ = 1.1 Co_FCC_. The replacement of Co with Ni in the AlCoCrFeNi alloy was proposed to not only effectively reduce cost but also maintain the B2 matrix of this alloy. Therefore, this study discusses the effects of replacing Co with Ni on the microstructure, mechanical properties, and age hardening of AlCo_1−x_CrFeNi_1+x_ high-entropy alloys.

## 2. Materials and Methods

Three high-entropy alloys of AlCo_1−x_CrFeNi_1+x_ (x is the molar ratio; x = 0, 0.5, 1, denoted as X_0_, X_0.5_, and X_1_, respectively) were synthesized by vacuum induction melting under argon, followed by casting in a ceramic shell mold. Commercial-grade elements of Al, Co, Cr, Fe, and Ni (99.5% purity) were used as raw materials. Table 1 lists the chemical compositions of the three alloys. The dimensions of the ingots were 58 × 58 × 118 mm^3^. They were cut into flakes with dimensions of 10 × 10 × 2 mm^3^ to investigate the crystal structure, microstructure, and hardness before and after aging. Flakes of the same size were subjected to aging at 500 to 1100 °C, with a temperature gap of 100 °C, and aging was conducted for 144 h, followed by water quenching. The crystal structure was examined by X-ray diffraction (XRD; D2 Phaser, Bruker, Billerica, MA, USA) using Cu K_α_ radiation at a scanning rate of 2°/min and a 2θ range of 20–100°. The microstructure was observed by scanning electron microscopy (SEM; S3400N, Hitachi High-Tech Corporation, Tokyo, Japan) and transmission electron microscopy (TEM; Tecnai™ G2 F20, FEI Technologies Inc., Hillsboro, OR, USA). Samples for SEM examination were polished by using an Al_2_O_3_ suspension, followed by etching using aqua regia. The thin-foil TEM sample was prepared by twin-jet electropolishing using 5% perchloric acid for further TEM analysis. The chemical composition of the alloys and different phases in the alloy were determined by energy-dispersive spectrometry (EDS; XFlash^®^ 6|100, Bruker, Billerica, MA, USA; Oxford X-Max 80, Oxford Instruments, Abingdon, UK). The volume fraction of each phase in the as-cast alloys was calculated with the image analysis software (MDS-Pro, FIRST OPTO-TECHNOLOGY CO., Kaohsiung, Taiwan). The Vickers hardness tester (FM-300e, Future-Tech Corp., Kawasaki, Japan) was used to measure hardness under a load of 1 kg for a duration of 15 s. Ten locations were measured to calculate the average hardness and standard deviation. Compression tests were conducted by using a universal tester (CY-6040A4, Chun-Yen Testing Machines Co., Taichung, Taiwan) under a strain rate of 10^−3^ s^−1^. For the compression test, the specimen size was ϕ5 × 10 mm^2^.

## 3. Results and Discussion

### 3.1. The As-Cast Alloys

#### 3.1.1. Crystal Structure

**Figure 1:**
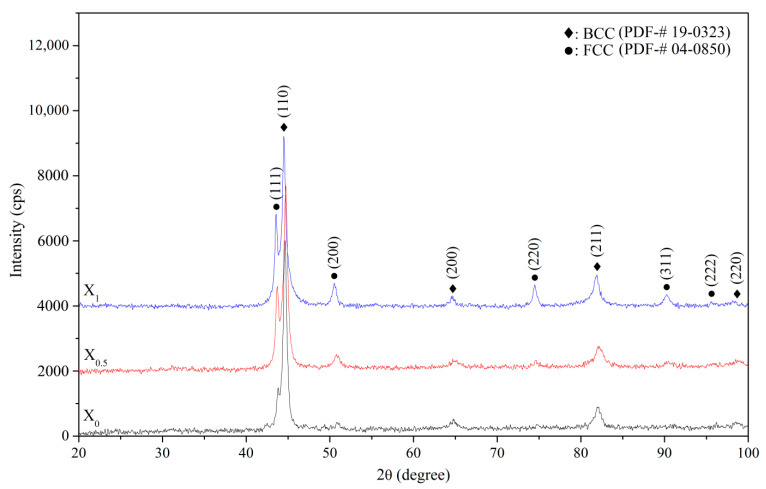
XRD patterns of the X_0_, X_0.5_, and X_1_ as-cast alloys.

Figure 1 shows XRD patterns of the X_0_, X_0.5_, and X_1_ as-cast alloys. Two phases were identified in the as-cast alloys, i.e., BCC (a = 2.865 Å) and FCC (a = 3.579 Å) phases. The BCC and FCC volume fractions were quantified by XRD with Cu K_α_ radiation via direct comparison [21], which uses the integration of the most intense peaks for the BCC phase (characterized by the (110), (200), (211), and (220) planes) and FCC phase (characterized by the (111), (200), (220), and (311) planes). Origin™ software was used for integrating these peaks with a peak-fitting tool. The volume fractions of the FCC phase for X_0_, X_0.5_, and X_1_ alloys were 7.7, 23.0, and 31.9%, respectively, indicating that compared with Co, Ni is a stronger FCC stabilizer. The FCC phase detected for the as-cast X_0_ alloy is speculative, because the cooling rate (10^−1^ to 10^−2^ K/s) of the melt in the ceramic shell mold by vacuum induction melting is lower than that (10–20 K/s) of the water-cooled copper mold by vacuum arc re-melting, as reported in previous studies [3,4,5,6,7,8,9,10,11,12,13,14]. The fact that the FCC phase of as-cast AlCoCrFeNi is formed after homogenization at 1100 °C [13] confirms this hypothesis.

#### 3.1.2. Microstructure

**Figure 2:**
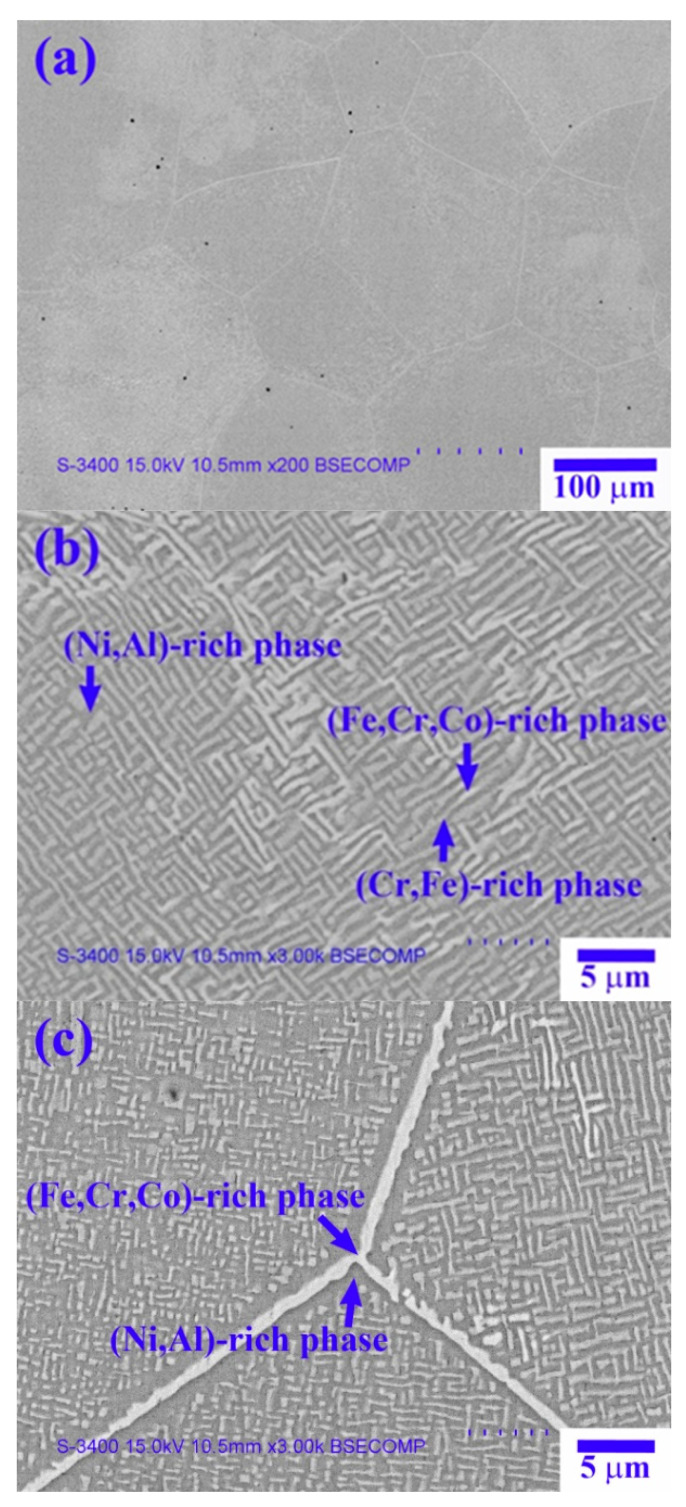
SEM-BSE images of the X_0_ as-cast alloy: (**a**) low magnification; (**b**) high magnification of dendrite; and (**c**) high magnification of interdendrite.

Figure 2 shows SEM-backscattered electron (BSE) images of the X_0_ as-cast alloy. Table 2 lists the chemical composition and volume fraction of the interior phases in Figure 2, as analyzed by EDS and image analysis, respectively. The low-magnification image shown in Figure 2a reveals the dendrite cell structure of the X_0_ as-cast alloy, with a cell size of 100–250 µm. Figure 2b shows the high-magnification image of the dendrite region, which has a spinodal decomposition (SD) structure comprising a dark-gray (Ni,Al)-rich matrix + light-gray wall-shaped (Cr,Fe)-rich phase [13], as well as a bright wall-shaped (Fe,Cr,Co)-rich phase. The interdendrite region comprises a dark-gray (Ni,Al)-rich matrix and a bright boundary-like (Fe,Cr,Co)-rich phase (Figure 2c).

**Figure 3:**
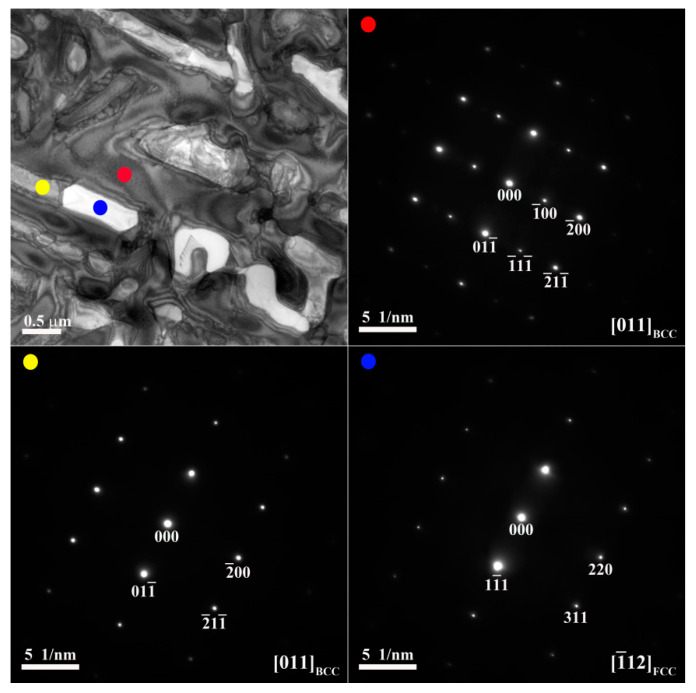
TEM bright-field images and SAED patterns of the dendrite for the X_0_ as-cast alloy (red dot: ordered BCC; yellow dot: BCC; blue dot: FCC).

**Figure 4:**
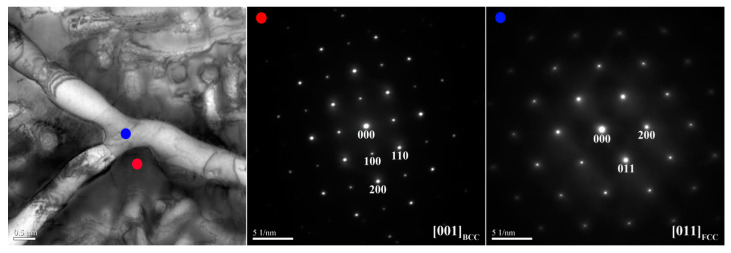
TEM bright-field images and SAED patterns of the interdendrite for the X_0_ as-cast alloy (red dot: ordered BCC; blue dot: FCC).

Figure 3 and Figure 4 show TEM bright-field images and selected-area electron diffraction (SAED) patterns of the dendrite and interdendrite in the X_0_ alloy, respectively. Table 3 lists chemical compositions of the interior phases marked in Figure 3 and Figure 4. The SAED and EDS analysis revealed the presence of the dark-gray (Al,Ni)-rich ordered BCC (B2) phase, light-gray wall-shaped (Cr,Fe)-rich BCC phase, and bright, wall-shaped (Fe,Cr,Co)-rich FCC phase (Figure 3 and Figure 4). Notably, in the dendrite region, the BCC (yellow dot) and FCC (blue dot) phases formed in one wall-shaped structure (Figure 3), indicating that the FCC phase transformed from the BCC phase because both phases are rich in Fe, Cr, and Co, and are deficient in Ni and Al.

**Figure 5:**
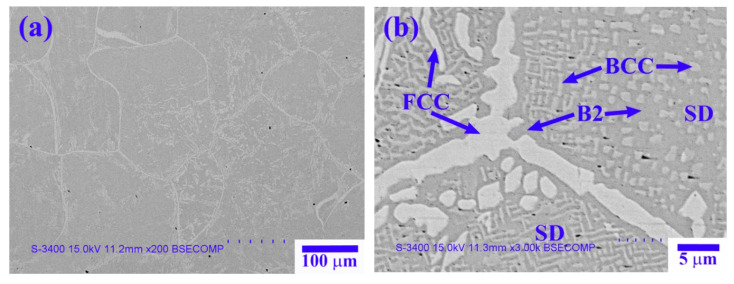
SEM-BSE images of the X_0.5_ as-cast alloy: (**a**) low magnification; (**b**) high magnification.

Figure 5 shows SEM-BSE images of the X_0.5_ as-cast alloy, and Table 4 lists the chemical composition and volume fraction of the interior phases. Figure 5a shows the microstructure of the X_0.5_ as-cast alloy: a dendritic cell structure with a cell size of 120–300 µm is observed. The high-magnification image shown in Figure 5b of the dendrite region indicated that it was constructed by an SD structure (dark-gray (Ni,Al)-rich B2 matrix + light-gray wall-shaped or spherical (Cr,Fe)-rich BCC phase) and a bright rod-like (Fe,Cr,Ni)-rich FCC phase. The interdendrite region comprises the dark-gray (Ni, Al)-rich B2 matrix and a bright boundary-like (Fe,Cr,Ni)-rich FCC phase (Figure 5b). Compared with the X_0_ as-cast alloy, the wall-shaped BCC phase exhibits spheroidization in the center region of the dendrite, and the FCC phase coarsens to a rod-like structure. In the interdendrite, the FCC phase is branched into the dendrite region. In addition, the replacement of Co with Ni permits the transformation of the (Fe,Cr,Co)-rich FCC phase in the X_0_ alloy to the (Fe,Cr,Ni)-rich phase in the X_0.5_ alloy.

**Figure 6:**
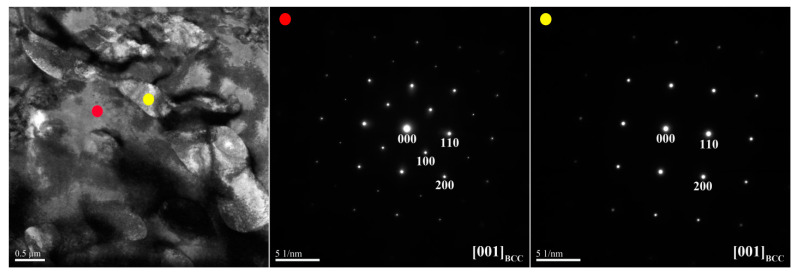
TEM bright-field image and SAED patterns of the dendrite for the X_0.5_ as-cast alloy (red dot: ordered BCC; yellow dot: BCC).

**Figure 7:**
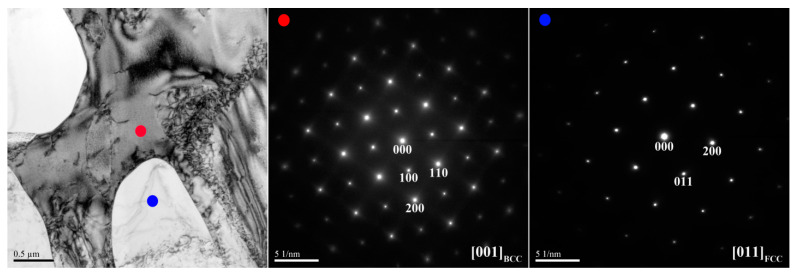
TEM bright-field image and SAED patterns of the interdendrite for the X_0.5_ as-cast alloy (red dot: ordered BCC; blue dot: FCC).

Figure 6 and Figure 7 show TEM bright-field images and SAED patterns of the X_0.5_ as-cast alloy, respectively. Table 5 lists the chemical composition of interior phases marked in Figure 6 and Figure 7. Figure 6 shows the SD structure in the center region of the dendrite. This structure is a combination of the (Ni,Al)-rich B2 matrix and (Cr,Fe)-rich BCC phase. Figure 7 shows the assembly of the interdendrite by the (Ni,Al)-rich B2 matrix and the (Fe,Cr,Ni)-rich FCC phase.

**Figure 8:**
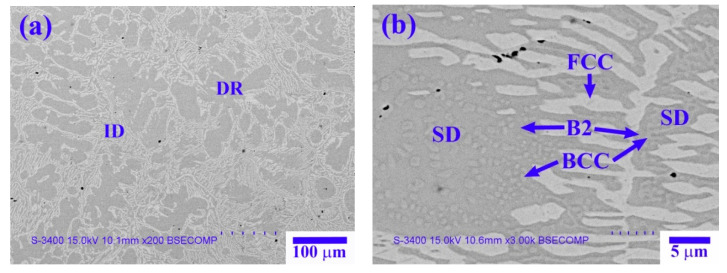
SEM-BSE images of the X_1_ as-cast alloy: (**a**) low magnification; (**b**) high magnification.

**Figure 9:**
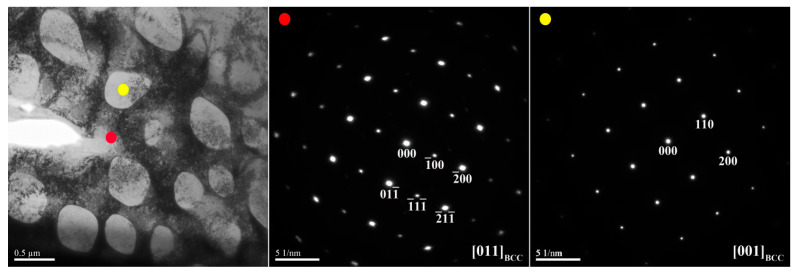
TEM bright-field image and SAED patterns of dendrite for the X_1_ as-cast alloy (red dot: ordered BCC; yellow dot: BCC).

**Figure 10:**
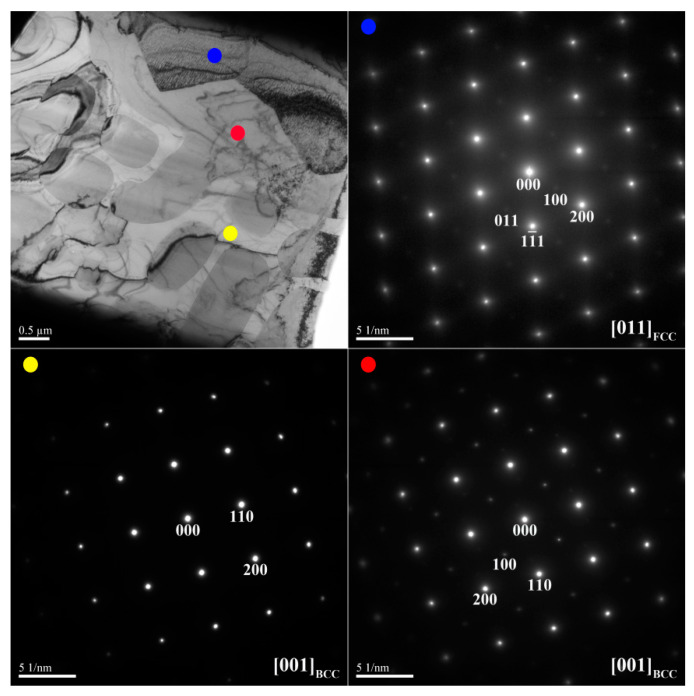
TEM bright-field image and SAED patterns of the interdendrite for the X_1_ as-cast alloy (red dot: ordered BCC; yellow dot: BCC; blue dot: FCC).

Figure 8 shows the microstructure of the X_1_ as-cast alloy, and Table 6 lists the chemical composition and volume fraction of the interior phases marked in Figure 8. The X_1_ as-cast alloy exhibited a dendritic structure (Figure 8a). The high-magnification image of the SD structure shown in Figure 8b reveals a dendrite region (DR) comprising a dark-gray (Ni,Al)-rich B2 matrix and a light-gray spherical (Cr,Fe)-rich BCC phase. The interdendrite (ID) region comprises a lamellar (Ni,Fe,Cr)-rich FCC phase and an SD structure between the FCC phase. Figure 9 and Figure 10 show TEM bright-field images and SAED patterns, respectively, of the X_1_ as-cast alloy. Table 7 lists chemical composition of the interior phases marked in Figure 9 and Figure 10. The SAED patterns and EDS results shown in Figure 9 and Figure 10 confirm that the dendrite comprises (Ni,Al)-rich B2 and (Cr,Fe)-rich BCC phases. The interdendrite comprises (Ni,Al)-rich B2 and (Ni,Fe,Cr)-rich FCC phases.

Comparison of the X_0.5_ and X_1_ as-cast alloys revealed that the chemical composition of the FCC phase in the interdendrite transformed from the (Fe,Cr,Ni)-rich phase in the X_0.5_ alloy to the (Ni,Fe,Cr)-rich phase in the X_1_ alloy because of the complete substitution of Co with Ni. Furthermore, the SAED pattern of the (Ni,Fe,Cr)-rich FCC phase shown in Figure 10 reveals an ordered phase. With respect to the phase transformation theory [22], in systems with strong chemical bonding between the atoms, ordered phases tend to be formed. The negative mixing enthalpy (ΔH_mix_) between Ni and the other constitutional elements is thought to increase in comparison with that between those elements and Co [23]; hence, the number of stronger Ni–Al, Ni–Cr, and Ni–Fe bonds increases, also further increasing with the substitution of Co with Ni in the alloy. Hence, the X_1_ alloy exhibited an ordered FCC phase. The volume fractions of the FCC phase in X_0_, X_0.5_, and X_1_ alloys calculated by image analysis were close to the results calculated by XRD peaks.

#### 3.1.3. Mechanical Properties

**Figure 11:**
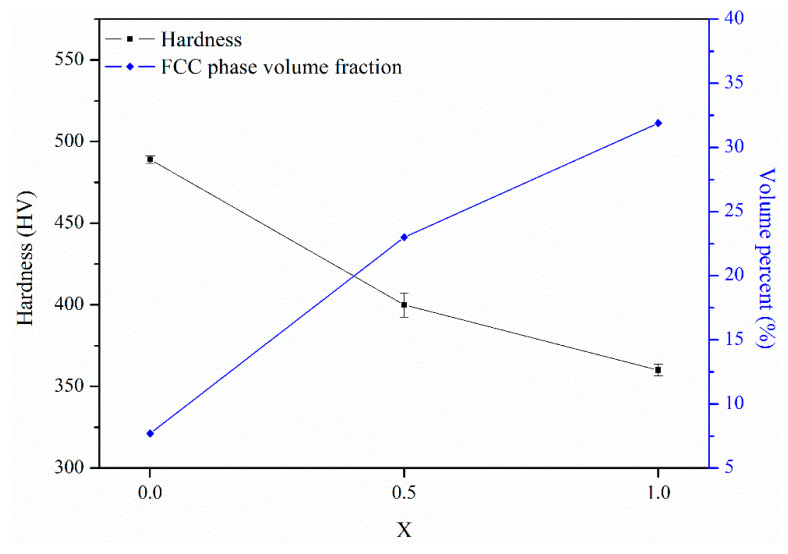
Variations in the hardness and FCC phase volume fraction of the X_0_, X_0.5_, and X_1_ as-cast alloys.

**Figure 12:**
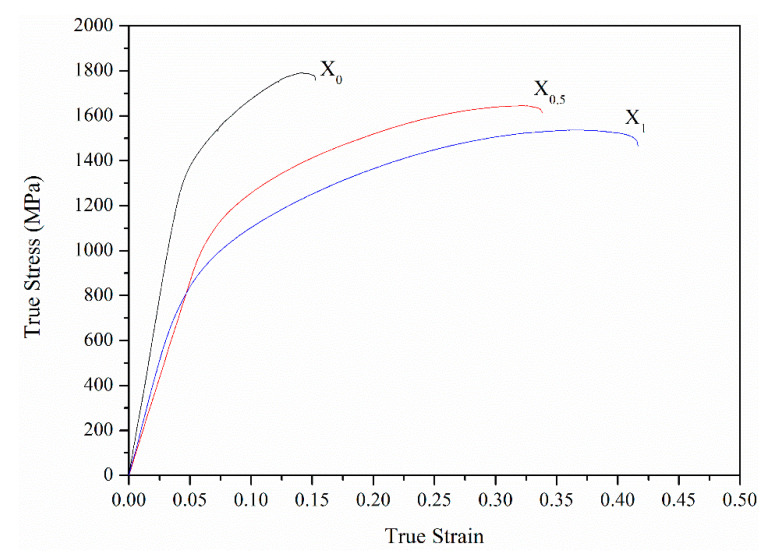
True stress–strain curves obtained from compression tests for the X_0_, X_0.5_, and X_1_ as-cast alloys.

Figure 11 shows variations in the hardness and FCC phase volume fraction for the X_0_, X_0.5_, and X_1_ as-cast alloys. From the substitution of Co with Ni, the hardness decreased by 25% from HV480 to HV360. Meanwhile, the FCC phase volume fraction increased from 7.7 to 31.9%. The decrease in the hardness was caused by the increase in the volume fraction of the soft FCC phase. From a previous report [13], the hardness of as-cast AlCoCrFeNi alloy is HV484, which is close to the present X_0_ alloy. Figure 12 shows compressive true stress–strain curves of the X_0_, X_0.5_, and X_1_ as-cast alloys, and Table 8 lists yield stress (σ_y_), compressive stress (σ_max_), and fracture strain (ε_f_) of the three alloys. From X_0_ to X_1_ alloys, the σ_y_ and σ_max_ decreased from 1202 and 1790 MPa to 693 and 1537 MPa, respectively. However, ε_f_ increased from 0.15 to 0.42. This result is also related to the increase in the amount of the soft FCC phase. Notably, from the X_0_ to X_1_ alloy, σ_max_ slightly decreased by 14%, but ε_f_ considerably increased by 180%, indicating that the substitution of Co with Ni can effectively improve the strength-ductility balance. Comparing the compressive properties of the X_0_ alloy with AlCrFeCoNi alloy mentioned in the Introduction, the X_0_ alloy exhibited lower σ_max_ and ε_f_. We suggest that the worse cleanliness of the present alloy synthesized by the vacuum induction melting process under Al_2_O_3_ crucible than previously published AlCrFeCoNi alloy [15] synthesized by vacuum arc re-melting process is the main reason.

**Figure 13:**
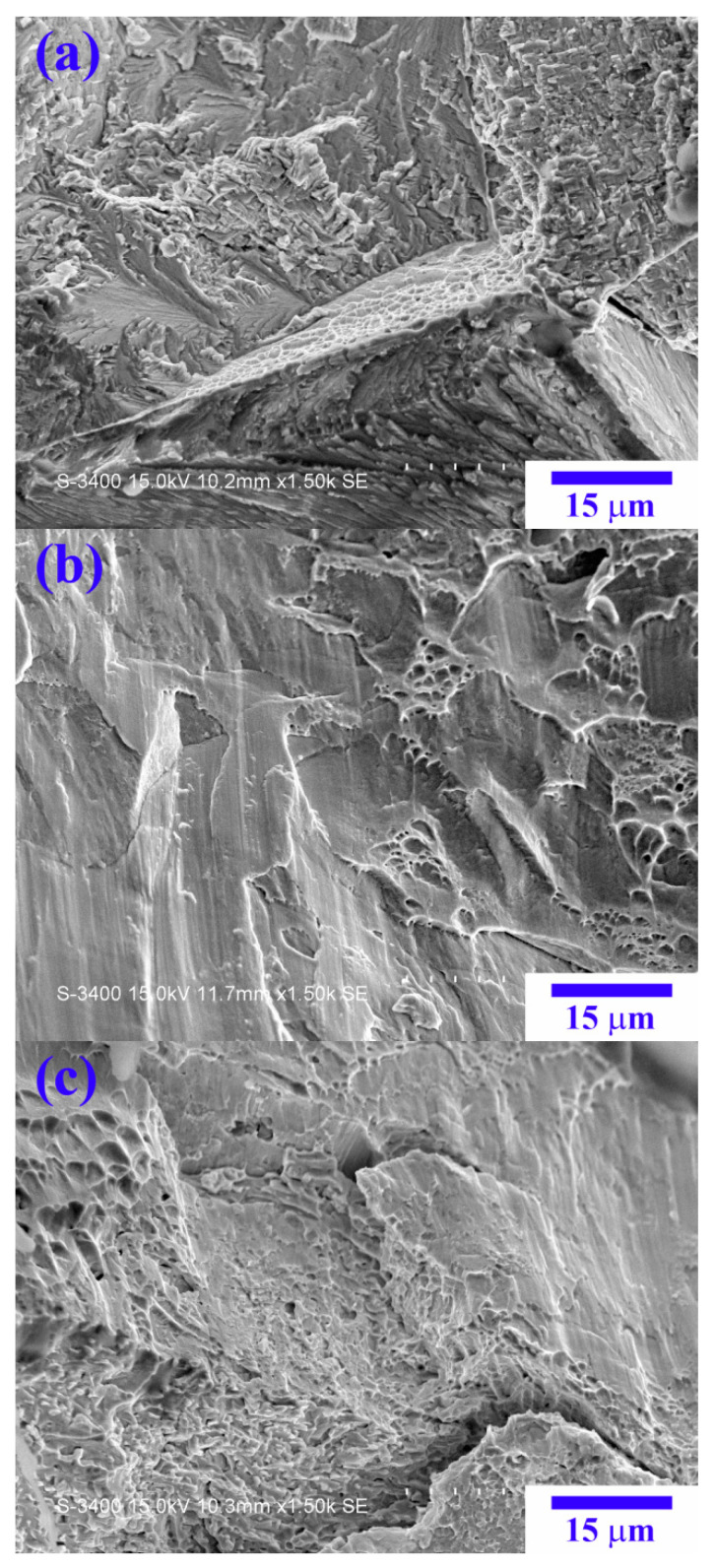
SEM images of fracture surfaces of the (**a**) X_0_; (**b**) X_0.5_; and (**c**) X_1_ alloys after compressive test.

Figure 13 shows SEM images of the fracture surfaces after compression. From the X_0_ to X_1_ alloy, the amounts of the dimple structure clearly increase, indicative of the increase in the alloy ductility by the replacement of Co by Ni.

### 3.2. The Aged Alloys

#### 3.2.1. Variations in Hardness and Crystal Structure

**Figure 14:**
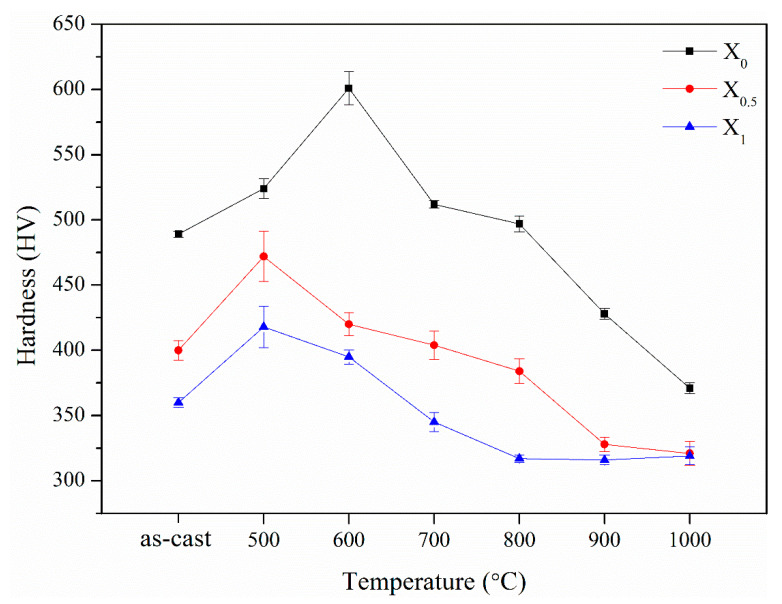
Variations in the hardness of the X_0_, X_0.5_, and X_1_ as-cast alloys after aging for 144 h at 500–1000 °C.

Figure 14 shows variations in the hardness for the X_0_, X_0.5_, and X_1_ aged alloys at 500–1000 °C. For the X_0_ alloy, with the increase in the aging temperature, the hardness increased from HV480 to a peak value of HV601 (25% increase) at 600 °C, followed by a decrease with the increase in the aging temperature to the bottom value HV371 at 1000 °C. The peak hardness with the increase in the aging temperature for the X_0.5_ and X_1_ alloys was observed at 500 °C for HV472 (18% increase) and HV418 (16% increase), respectively. At 600–1000 °C, with the increase in the aging temperature, the hardness of the X_0.5_ and X_1_ alloys decreased to the bottom values of HV318 and HV 320, respectively. The temperature ranges for age hardening for the X_0_, X_0.5_, and X_1_ alloys were 500–800, 500–700, and 500–600 °C, respectively. Moreover, age softening was observed at 900–1000, 800–1000, and 700–1000 °C for the X_0_, X_0.5_, and X_1_ alloys, respectively. The following crystal and microstructure analyses describe the aging behaviors.

**Figure 15:**
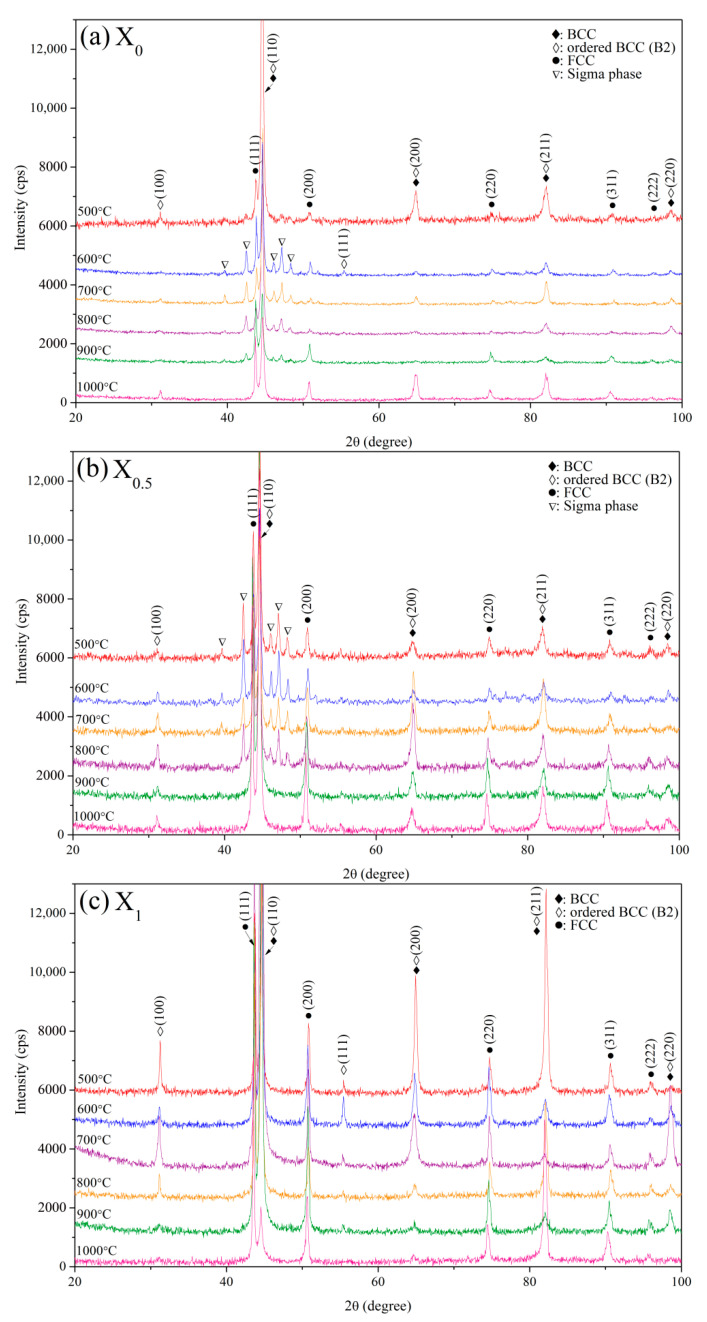
XRD patterns of the three as-cast alloys after aging at 500–1000 °C for 144 h: (**a**) X_0_; (**b**) X_0.5_; and (**c**) X_1_.

Figure 15 shows XRD patterns of the three aged alloys. The three aged alloys were primarily composed of the B2, BCC, and FCC phases. The Cr,Fe-like σ phase (a = 8.800 Å, c = 4.544 Å, c/a = 0.516, PDF-# 05-0708) was identified in the X_0_ and X_0.5_ alloys at 500–900 °C and 500–800 °C, respectively. For the X_0_ alloy, from the diffraction peak intensity, the volume fraction of the σ phase increased with the increase in the temperature from 500 to 600 °C and decreased from 700 to 900 °C (Figure 15a). With the increase in temperature from 700 to 1000 °C, the volume fraction of the FCC phase increases. The maximum amount of the σ phase formed at 500 °C and decreased from 600 to 800 °C for the X_0.5_ alloy (Figure 15b). Similar to the X_0_ alloy, the volume fraction of the FCC phase increased with the increase in temperature from 700 to 1000 °C. The maximum amount of the BCC phase was generated at 500 °C for the X_1_ alloy (Figure 15c). The variation in the FCC phase for the X_1_ alloy was similar to that for the X_0_ and X_0.5_ alloys, which increased with the increase in temperature from 700 to 1000 °C. Figure 14 shows the age hardening corresponding to the formation of the σ phase in X_0_ and X_0.5_ alloys and the BCC phase in the X_1_ alloy. Furthermore, the increase in the amount of the FCC phase led to age softening for the X_0_, X_0.5_, and X_1_ alloys. Analysis of the aging behavior of these three alloys revealed that the replacement of Co with Ni inhibits the formation of the σ phase and a weak hardening effect, i.e., the effect of peak age hardening decreases from 25 to 16% from x = 0 to x = 1. In previous reports [7,13,24], the hardness of σ, BCC, and FCC phases in HEAs were HV660, HV484, and HV208, respectively.

#### 3.2.2. Microstructure

**Figure 16:**
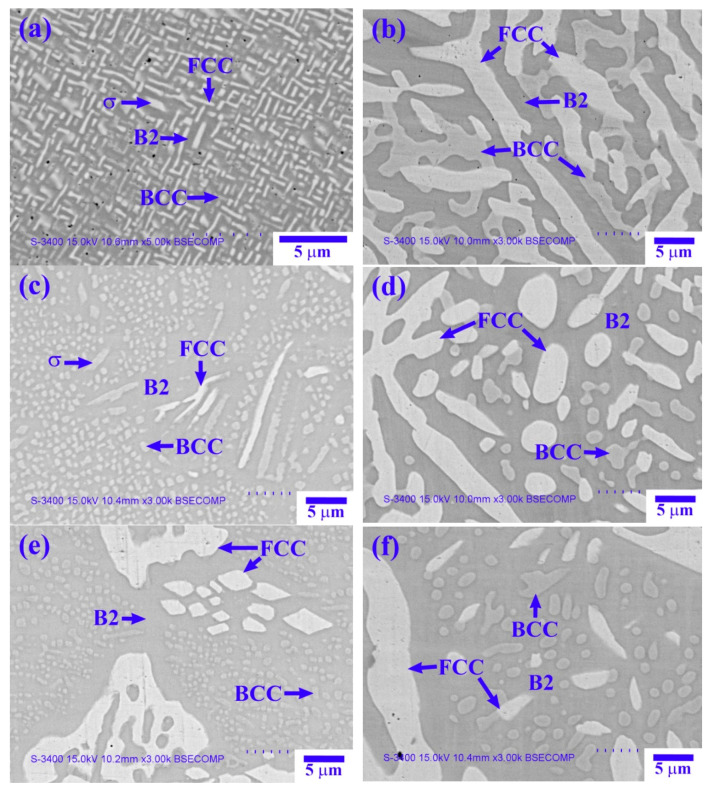
SEM-BSE images of the three aged alloys: (**a**,**b**) X_0_ at 600 and 1000 °C; (**c**,**d**) X_0.5_ at 500 and 1000 °C; and (**e**,**f**) X_1_ at 500 and 1000 °C.

Figure 16 shows SEM-BSE images of the three aged alloys, and Table 9 lists chemical compositions of the interior structures marked in Figure 16. The results in Table 9 reveal that the σ phase in the aged alloys was a (Cr,Fe)-rich phase. The chemical composition characteristics of the B2, BCC, and FCC phases in the aged alloys were the same as those of the as-cast alloy, i.e., the (Ni,Al)-rich phase, (Cr,Fe)-rich phase, and (Fe,Cr,Co)-rich/(Fe,Cr,Ni)-rich or (Ni,Fe,Cr)-rich phase. The precipitation of the blocky σ phase from the matrix at 600 °C for the X_0_ alloy caused peak age hardening (Figure 16a). The σ phase completely dissolved into the B2 matrix, and the BCC and FCC phases underwent coarsening at 1000 °C, leading to bottom age softening (Figure 16b). The precipitation of the blocky σ phase from the matrix at 500 °C for the X_0.5_ alloy caused peak age hardening (Figure 16c). In Figure 16d, it can be seen that the disappearance of the σ phase and coarsening of BCC and FCC phases led to bottom age softening at 1000 °C. Comparison of Figure 8b with Figure 16e reveals that Figure 16e shows the precipitation of fine and dense BCC particles from the B2 matrix, which led to peak age hardening at 500 °C. The coarsening of the BCC and FCC phases led to bottom age softening at 1000 °C (Figure 16f).

## 4. Conclusions

Three as-cast HEAs of AlCo_1−x_CoFeNi_1+x_ (x is the molar ratio, where x = 0, 0.5, 1, denoted as X_0_, X_0.5_, and X_1_, respectively) exhibited a dendritic structure. The dendrites of the X_0_, X_0.5_, and X_1_ alloys comprised the B2 + BCC + FCC phases, B2 + BCC phases, and B2 + BCC phases, respectively. The interdendrite of the X_0_, X_0.5_, and X_1_ alloys comprised the B2 + FCC phases, B2 + FCC phases, and B2 + BCC + ordered FCC phases, respectively. By the replacement of Co with Ni, the hardness decreased from HV480 to HV360, while the FCC phase volume fraction increased from 7.7 to 31.9%. From X_0_ to X_1_ alloys, the σ_y_ and σ_max_ decreased from 1202 and 1790 MPa to 693 and 1537 MPa, respectively. However, ε_f_ increased from 0.15 to 0.42. For the X_0_ alloy, peak age hardening occurred at 600 °C because of the precipitation of the σ phase. The peak age hardening for the X_0.5_ and X_1_ alloys, which was caused by the precipitation of the σ and BCC phases, respectively, was observed at 500 °C. Age softening for the X_0_, X_0.5_, and X_1_ alloys was observed at 900–1000 °C, 800–1000 °C, and 700–1000 °C, respectively, because of the increase in the amount of the FCC phase and coarsening of the BCC phase. The replacement of Co with Ni effectively improved the strength–ductility balance.

## Figures and Tables

**Table 1:** Chemical composition (at.%) of the X_0_, X_0.5_, and X_1_ alloys.

Alloy	Al	Co	Cr	Fe	Ni
X_0_	21.1	21.2	18.7	18.7	20.3
X_0.5_	20.6	11.2	18.8	18.8	30.6
X_1_	22.3	N/A	18.0	19.2	40.5

**Table 2:** Chemical composition (at.%) and volume fraction (%) of the interior phases marked in Figure 2.

Phase	Volume Fraction	Al	Co	Cr	Fe	Ni
(Ni,Al)-rich phase	62.7 ± 2.1	31.2 ± 2.8	20.4 ± 1.6	8.2 ± 0.9	13.6 ± 1.7	26.6 ± 1.1
(Cr,Fe)-rich phase	27.2 ± 2.3	11.5 ± 2.0	19.3 ± 1.1	35.8 ± 2.5	23.6 ± 1.3	9.8 ± 0.6
(Fe,Cr,Co)-rich phase	10.1 ± 1.1	7.4 ± 1.1	25.4 ± 1.5	25.2 ± 2.3	26.8 ± 1.4	15.2 ± 1.8

**Table 3:** Chemical composition (at.%) of the interior phases marked in Figure 3 and Figure 4.

Region	Phase	Al	Co	Cr	Fe	Ni
Dendrite	B2	24.7 ± 1.9	21.8 ± 0.5	6.0 ± 1.3	15.9 ± 1.4	31.6 ± 2.3
	BCC	2.3 ± 1.1	15.9 ± 1.1	49.4 ± 3.9	27.2 ± 1.1	5.2 ± 0.4
	FCC	3.2 ± 1.4	26.4 ± 1.2	27.0 ± 1.5	31.0 ± 1.2	12.4 ± 1.6
Interdendrite	B2	24.5 ± 1.8	21.5 ± 1.6	7.0 ± 0.3	15.0 ± 1.7	32.0 ± 1.9
	FCC	3.8 ± 1.7	24.6 ± 1.2	28.6 ± 1.4	29.6 ± 1.5	13.4 ± 1.1

**Table 4:** Chemical composition (at.%) and volume fraction (%) of the interior phases marked in Figure 5.

Phase	Volume Fraction	Al	Co	Cr	Fe	Ni
B2	52.6 ± 2.1	33.8 ± 2.7	9.7 ± 0.8	5.5 ± 1.0	12.2 ± 1.4	38.8 ± 3.1
BCC	24.1 ± 1.7	1.4 ± 0.6	13.1 ± 1.5	47.3 ± 1.9	30.7 ± 1.9	7.5 ± 0.4
FCC	23.3 ± 1.4	7.9 ± 1.9	14.1 ± 1.1	26.0 ± 1.7	27.6 ± 1.4	24.4 ± 1.6

**Table 5:** Chemical composition (at.%) of the interior phases marked in Figure 6 and Figure 7.

Region	Phase	Al	Co	Cr	Fe	Ni
Dendrite	B2	34.3 ± 2.1	8.7 ± 1.5	2.8 ± 1.0	11.9 ± 1.7	42.3 ± 3.4
	BCC	1.0 ± 0.7	10.3 ± 1.4	53.1 ± 2.5	30.8 ± 1.6	4.8 ± 1.4
Interdendrite	B2	33.1 ± 2.6	8.8 ± 1.6	3.4 ± 0.4	11.2 ± 0.8	43.5 ± 2.2
	FCC	5.7 ± 1.1	12.9 ± 1.8	28.9 ± 1.9	30.2 ± 2.3	22.3 ± 1.3

**Table 6:** Chemical composition (at.%) and volume fraction (%) of the interior phases marked in Figure 8.

Phase	Volume Fraction	Al	Co	Cr	Fe	Ni
B2	49.0 ± 2.3	34.5 ± 2.3	N/A	4.6 ± 0.8	11.2 ± 0.6	49.7 ± 2.7
BCC	18.2 ± 1.5	4.5 ± 0.6	N/A	45.4 ± 2.1	30.5 ± 2.3	19.6 ± 1.9
FCC	32.8 ± 1.9	7.5 ± 1.2	N/A	27.5 ± 1.6	30.4 ± 1.5	34.6 ± 2.1

**Table 7:** Chemical composition (at.%) of the interior phases marked in Figure 9 and Figure 10.

Region	Phase	Al	Co	Cr	Fe	Ni
Dendrite	B2	33.9 ± 2.4	N/A	3.0 ± 0.5	10.3 ± 0.7	52.8 ± 2.5
	BCC	0.9 ± 0.4	N/A	63.0 ± 2.7	30.2 ± 1.1	5.9 ± 1.0
Interdendrite	B2	33.5 ± 1.5	N/A	2.8 ± 0.6	11.6 ± 1.2	52.1 ± 2.2
	BCC	0.9 ± 0.7	N/A	62.3 ± 2.4	30.3 ± 1.4	6.5 ± 1.2
	FCC	7.0 ± 1.3	N/A	28.4 ± 1.6	30.3 ± 1.9	34.3 ± 1.8

**Table 8:** Yield stress (σ_y_), compressive stress (σ_max_), and fracture strain (ε_f_) of the X_0_, X_0.5_, and X_1_ as-cast alloys.

Alloy	σ_y_ (MPa)	σ_max_ (MPa)	ε_f_
X_0_	1202	1790	0.15
X_0.5_	989	1644	0.33
X_1_	653	1537	0.42

**Table 9:** Chemical composition (wt.%) of the interior structures marked in Figure 16.

Alloy	Phase	Al	Co	Cr	Fe	Ni
X_0_-600 °C	B2	26.8 ± 1.3	20.8 ± 1.4	12.7 ± 1.3	15.6 ± 1.9	24.2 ± 1.3
	BCC	14.7 ± 1.5	22.1 ± 1.2	27.7 ± 1.7	21.9 ± 1.2	13.6 ± 1.5
	σ	7.6 ± 1.9	21.4 ± 1.8	37.8 ± 2.9	25.3 ± 1.7	7.7 ± 1.7
	FCC	8.6 ± 0.7	25.8 ± 1.7	23.0 ± 1.1	28.3 ± 1.4	14.3 ± 1.1
X_0_-1000 °C	B2	32.1 ± 2.4	19.6 ± 1.2	8.2 ± 0.4	11.7 ± 1.1	28.5 ± 1.8
	BCC	6.8 ± 0.6	20.3 ± 1.1	38.5 ± 2.4	25.2 ± 0.7	9.2 ± 1.1
	FCC	6.7 ± 1.0	24.9 ± 1.0	26.6 ± 1.4	27.4 ± 1.2	14.4 ± 0.6
X_0.5_-500 °C	B2	32.5 ± 2.1	8.0 ± 1.0	6.0 ± 0.8	12.2 ± 1.0	41.3 ± 1.5
	BCC	8.8 ± 1.1	17.5 ± 1.6	33.6 ± 1.2	26.7 ± 1.6	13.4 ± 1.4
	σ	4.6 ± 1.4	11.7 ± 1.3	41.8 ± 3.1	29.5 ± 1.5	12.4 ± 1.1
	FCC	7.9 ± 1.3	15.0 ± 1.8	24.4 ± 1.9	27.9 ± 0.7	24.8 ± 1.6
X_0.5_-1000 °C	B2	32.1 ± 1.8	7.8 ± 1.6	6.2 ± 1.1	12.4 ± 1.4	41.5 ± 2.8
	BCC	9.3 ± 1.1	16.2 ± 1.9	34.0 ± 1.3	26.9 ± 1.1	13.6 ± 0.4
	FCC	7.1 ± 1.2	14.1 ± 1.2	25.3 ± 1.7	28.6 ± 1.9	24.9 ± 1.9
X_1_-500 °C	B2	29.5 ± 1.6	N/A	5.3 ± 0.9	10.8 ± 1.0	54.3 ± 2.5
	BCC	8.8 ± 0.7	N/A	50.1 ± 2.0	19.6 ± 1.3	21.5 ± 1.3
	FCC	6.0 ± 0.9	N/A	27.0 ± 1.5	31.2 ± 2.1	35.9 ± 1.6
X_1_-1000 °C	B2	29.5 ± 2.1	N/A	6.6 ± 0.5	10.8 ± 1.5	53.2 ± 1.5
	BCC	9.0 ± 1.5	N/A	53.2 ± 2.2	19.4 ± 1.7	18.4 ± 1.2
	FCC	6.6 ± 1.7	N/A	26.4 ± 1.4	30.7 ± 1.1	36.3 ± 2.6

## Data Availability

Data sharing not applicable.
